# Current Challenges for Early Career Researchers in Academic Research Careers: COVID‐19 and Beyond

**DOI:** 10.1002/jbm4.10540

**Published:** 2021-08-19

**Authors:** Rachelle W. Johnson, Megan M. Weivoda

**Affiliations:** ^1^ Division of Clinical Pharmacology, Department of Medicine Vanderbilt University Medical Center Nashville TN USA; ^2^ Division of Clinical Pharmacology, Department of Medicine, Vanderbilt Center for Bone Biology Vanderbilt University Medical Center Nashville TN USA; ^3^ Periodontics and Oral Medicine University of Michigan School of Dentistry Ann Arbor MI USA; ^4^ Biointerfaces Institute University of Michigan Ann Arbor MI USA

The academic landscape has changed extensively over the past few decades for early career researchers (ECRs), defined as those within approximately the first 10 years of earning their terminal degrees. Some of these changes are for the better. There is increased awareness of unconscious bias and its impact on hiring, salary, and promotions. There is less tolerance for bullying and harassment, and greater emphasis on mental health and well‐being. However, there are new obstacles facing today's ECRs as they strive for independent careers in academia. There is reduced availability of tenure‐track faculty positions relative to the number of PhD graduates; institutional support is increasingly moving toward soft‐money (positions supported in large part or wholly by extramural grant funding); and funding rates by granting institutions are lower as increases in budget fail to keep up with a growing pool of applicants. And then came a global pandemic.

Not surprisingly, the past year‐and‐a‐half has been challenging for scientists at all career stages, because non‐essential lab work was temporarily halted and capacity restrictions severely limited the pace of research. Even with most institutions having now returned to pre‐pandemic capacity, research continues to be affected by backorders of essential laboratory products and reagents. However, ECRs have been uniquely impacted by Coronavirus Disease 2019 (COVID‐19). Graduate students and postdoctoral fellows were unable to perform experiments and face delays and potential lapses in funding that are beyond their control. Junior faculty in the process of starting new laboratories and establishing independent careers, a period in which productivity is pivotal for tenure and future academic security, faced hiring freezes, ordering and pipeline restrictions, and lack of a community in which to establish collaborations with departmental colleagues. On top of this, ECRs and other investigators with infants and school‐aged children suffered from the closures of daycares, schools, and the transition to virtual learning. A study published early in the pandemic reported that female scientists and researchers with children under 5 years reported the largest declines in time devoted to research during the pandemic,^(^ ^1^ ^)^ and the NIH recently reported that 63% of ECRs were concerned about the negative impact of the pandemic on their career trajectory.^(^ ^2^ ^)^ For ECRs impacted by COVID‐19 laboratory closings or the impacts on childcare, the opportunity for career growth was lost at a critical stage.

There have been silver linings, such as the ability to attend national and international conferences on virtual platforms that would otherwise have been financially or logistically impossible to attend, and more time with immediate family. These opportunities are of great value; however, the scale of a global pandemic cannot be downplayed. The stress of the pandemic on research productivity is amplified by the unique time in which ECRs are entering the academic arena to compete for independent funding. Funding rates in the United States remain far lower than they were a decade or two ago, when many of our senior colleagues were entering the field. This is not just perception; the NIH reported in 2014 that success rates, award rates, and funding rates were at their lowest in at least 25 years due to an increase in applicants and stagnation of NIH appropriations after 2003.^(^ ^3^ ^)^ It cannot be emphasized enough that the increase in grant applications over the past decade, without a comparable increase in allocated funding, has resulted in lower funding rates.

This is not to say that mid‐career or senior investigators do not face these same struggles for obtaining funding; they absolutely do. However, ECRs do not have a long track record of independent funding or publications and they frequently tread the fine line between proposing innovative, cutting‐edge science and being over‐ambitious. All scientists feel the pull of reviewer demands, which nearly always result in a stronger manuscript or grant application (even as we are silently cursing reviewer 3). At its best, our scientific approach is improved, but at its worst, an early career investigator may have a complete breakdown, not because they cannot handle multiple rounds of rejection, but because the collective stressors of this lifestyle are too much. In the case of ECRs that are on soft money, a gap in external funding can also result in the loss of a job. In this case it is important to re‐emphasize funding rates, and to note that an unfunded grant does not equate to bad science. How then can we prevent ECRs from exiting the academic pipeline, voluntarily or not, and, more provocatively, should we?

It is important for us to develop career‐related mindfulness in our trainees. What do they love about this career? What do they dislike? What are they passionate about? How might this translate into different careers within or outside academia? ECRs should not see their mentor's career as the only option for success; mentors should have these discussions with their mentees and encourage them to perform a self‐assessment at each career stage. ECRs should ask themselves whether they truly want to put that grant in again, and if so, what resources are needed? Expansion of financial support from the federal and institutional level and adjusted expectations from those in charge have been proposed as post‐COVID resources to bolster ECRs.^(^ ^4^ ^)^ Discussion around mental health resources should also be a cornerstone of these conversations, because securing a tenure‐track faculty position in academia requires a distillation of achievement and motivation that can easily lead to burnout. However, if the answer to this question is no, then ECRs should be supported by their mentors and member research societies in investigating alternative career paths. The shift of PhDs to nonacademic careers is in progress, and the number of PhDs in the private sector is now nearly equivalent to academia.^(^ ^5^ ^)^ Regardless of the path chosen, we need to elevate and support ECRs at all stages to ensure they have access to peer networks, supportive mentors, mental health resources, information about alternative career options, and appropriate career‐stage opportunities.

This special issue of *Journal of Bone and Mineral Research Plus*, which highlights work from the laboratories of ECRs, is one such opportunity. These investigators, representative of early career researchers at many different career stages and transitions and several of whom have recently started their laboratories, were selected for their outstanding contributions and commitment to the bone and mineral field. Despite the extreme challenges faced by ECRs, excellent research continues to be produced and this work should be recognized. We applaud the ECRs highlighted in this special issue for their efforts to perform high‐quality research while managing the challenges of COVID‐19 and other obstacles faced by ECRs.

## Conflict of Interest

The authors have no conflicts of interest to declare.

### Peer Review

The peer review history for this article is available at https://publons.com/publon/10.1002/jbm4.10540.
